# Assessment of Legibility and Completeness of Prescriptions at Tertiary Care Hospitals: A Cross-Sectional Study

**DOI:** 10.1192/bjo.2022.503

**Published:** 2022-06-20

**Authors:** Sajeel Saeed, Kashif Tousif, Tehseen Haider, Rubaid Azhar Dhillon, Mohammad Ebad ur Rehman, Attiya Munir, Omaima Asif, Nabeel Asif, Muhammad Arish

**Affiliations:** 1 Rawalpindi Medical University, Rawalpindi, Pakistan.; 2 Riphah International University, Rawalpindi, Pakistan

## Abstract

**Aims:**

The aim of this study was to assess the legibility as well as components of a prescription prescribed by doctors in tertiary care hospitals of Rawalpindi, Pakistan.

**Methods:**

An analytical cross-sectional study was conducted in pharmacies of two allied hospitals of Rawalpindi Medical University. Data were collected using stratified randomized sampling. A total of 661 prescriptions were selected and analysed for legibility by three experts. SPSS version 26.00 and Graph Pad Prism were used to enter and analyze the data. Descriptive statistics, correlational model and multinomial logistic regression were applied.

**Results:**

A total of 1982 drugs were prescribed in 661 prescriptions. A total of 46.0% prescriptions were classified in grade 2 and 32.1% in grade 3. On average, 55.74% prescriptions were found to be complete. On average, prescriber's information, patient's information and medication details were present in 72.64%, 57.25%, and 36.73% prescriptions, respectively. Grade 1 (AOR = 0.62), grade 2 (AOR = 0.83), and grade 3 (AOR = 0.85) prescriptions had less odds of being complete compared to grade 4 prescriptions.

**Conclusion:**

Majority of the prescriptions prescribed at tertiary care hospitals were barely legible and also quite a number of prescriptions were incomplete.

